# Impact of hepatitis C status on 20-year mortality of patients with substance use disorders

**DOI:** 10.1186/s13722-015-0041-6

**Published:** 2015-10-13

**Authors:** Anthony J. Accurso, Darius A. Rastegar, Sharon R. Ghazarian, Michael I. Fingerhood

**Affiliations:** Johns Hopkins Bayview Medical Center, 5200 Eastern Ave, Mason F. Lord Bldg, West Tower 5th floor, Baltimore, MD 21224 USA

**Keywords:** Hepatitis C virus, Chemical dependence, Survival

## Abstract

**Background:**

The magnitude of the effect of hepatitis C viral infection on survival is still not fully understood. The objective of this study was to determine whether the presence of hepatitis C viral antibodies in 1991 was associated with increased mortality 20 years later within a cohort of patients with substance use disorders. Secondary objectives were to determine other factors that were associated with increased mortality in the cohort.

**Methods:**

A subset of a 1991 study cohort of patients who had presented for detoxification was reexamined 20 years later. The Social Security Death Index was queried to identify which of the original patients had died. Attributes of survivors and non-survivors were compared, with special attention to their hepatitis C status in 1991. The original study and this analysis were conducted in the chemical detoxification unit at Johns Hopkins Bayview (previously Francis Scott Key Hospital), an academic urban hospital. All participants met the criteria for alcohol or opioid dependence at the time of admission in 1991. The primary study outcome was 20-year mortality after initial admission in 1991, with a planned analysis of hepatitis C status.

**Results:**

Twenty years after admission, 362 patients survived and 82 had died. Of the 284 patients who were hepatitis C positive, 228 survived (80 %). Of the 160 patients who were hepatitis C negative, 134 survived (84 %). This absolute risk increase of 4 % was not statistically significant (p = 0.37). Factors associated with increased mortality included male sex, white race, older age, and reported use of alcohol, cocaine, and illicit methadone. Binary logistic regression including hepatitis C status and these other variables yielded an adjusted odds ratio of 0.87 (95 % CI 0.49–1.55); (p = 0.64) for hepatitis C positive 20-year survival.

**Conclusions:**

Hepatitis C positivity was not associated with a statistically significant difference in 20-year survival. The effect of the virus on mortality, if present, is small, relative to the effect of substance use disorders alone.

## Background

Hepatitis C has been recognized as the major cause of chronic hepatitis in people who inject drugs since 1992 [1]. Epidemiologic studies have shown the prevalence of hepatitis C infection among drug users to be as high as 85–90 % in a variety of cities worldwide [2–4]. Most individuals who test positive for hepatitis C antibody have circulating hepatitis C virus, but 10–15 % clear the infection on their own [5]. It is estimated that about 50 % of infected individuals progress to chronic liver disease and 20 % progress to cirrhosis [6, 7]. The speed of progression appears multi-factorial, with progression accelerated by concurrent HIV infection and heavy alcohol use [8].

Since 1990, the treatment of hepatitis C has depended on the use of injectable interferon as the backbone of treatment [9, 10]. Related to the need for adherence, significant side effects and the intramuscular route of administration, a relatively small percentage of people who inject drugs have been considered for treatment, let alone treated. However, recent advances in the treatment of hepatitis C have thrust attention to the consideration of treatment for most individuals with hepatitis C viremia [11–15]. This attention is likely to continue to expand as more treatments without interferon are approved over the next few years.

Along with the expanding pharmacotherapy for hepatitis C, there has come the recommendation for expanded screening for hepatitis C [16–21]. The CDC has advocated for universal screening for hepatitis C for all Americans born between 1945 and 1964. This change in the screening guidelines stems from the fact that chronic hepatitis C infection can now be treated by several highly efficacious therapies [22, 23]. An important factor of these new treatment agents is their cost, ranging from 20 to 80 thousand dollars per treatment attempt [24]. Given the high cost of treatment, many third-party payers currently triage their patients based on the extent of liver disease and the likelihood of death from hepatitis C related illness. Knowledge of the natural history of hepatitis C in patients with substance use disorders can aid in the complex decision of who and when to treat. People who inject drugs have a heavy burden of chronic hepatitis C as a population, due to the viral mode of transmission, and are also known to have a higher risk of early mortality than the general population. The relative contribution of chronic hepatitis C infection in this population, compared to the contribution of other dangers such as overdose, suicide and homicide, is still not fully known [25]. Our study offers a prospective examination of this question.

In 1993, an early report on the prevalence of viral hepatitis C among alcohol and drug users showed the correlation of anti-HCV positivity with elevated liver enzymes in that population [1]. The cohort was found to have a hepatitis C antibody prevalence of 63 % overall, and 86 % for those who reported ever using an illicit drug by injection. The arrival of the twenty-year anniversary of this study prompted us to investigate the 20-year all-cause mortality of the cohort, with specific attention to the presence or absence of anti-HCV positivity in 1991. In this study, we look at mortality of a subset of the original cohort, with a focus on whether or not hepatitis C infection was associated with higher mortality.

## Methods

The original study was conducted on the Chemical Dependence Unit at Johns Hopkins Bayview Medical Center (then Francis Scott Key Medical Center), in Baltimore, Maryland, between November 1990 and May 1991. Then and now, the unit has 26 inpatient beds and serves a mostly indigent population in the city of Baltimore. Consecutive admissions were entered into the study. All patients met criteria for substance dependence. Demographic data were recorded and patient history related to substance use disorder, HIV infection, hepatitis and sexually transmitted infections was obtained. On admission to the unit, patients had blood testing which included tests for AST, ALT, hepatitis B surface antigen, hepatitis B surface antibody and rapid plasma regain (RPR) test for syphilis. All patients were offered HIV testing. The presence of hepatitis C antibody (anti-HCV) was determined by a first generation enzyme linked immunosorbent assay (Abbott Laboratories, Abbott Park, Illinois) that detects antibody to the C100-3 antigen. All positive serum samples were retested. Assays for hepatitis B were performed by enzyme immunoassay (Abbott Laboratories). The presence of HIV antibody was determined by an enzyme linked immunosorbent assay (Genetic Systems, Seattle, Washington) and all positive samples were confirmed by Western blot (Dupont Laboratories, Wilmington, Delaware).

Records from January 1991–May 1991 were located and 444 records were obtained. The Social Security Death Index was accessed to determine whether or not patients had died during the 20 years period since the date of admission. Causes of death were not determined. Prior to proceeding, anti-HCV status was chosen as a variable of interest in the data analysis. An initial bivariate analysis was performed on the mortality groups, using Chi-squared or Fisher’s Exact tests for categorical variables and independent groups t tests for continuous variables, with significance levels set at p < 0.05 using STATA 12.1. Anti-HCV status was then included in a regression analysis with all variables for which the mortality group comparison yielded p values of 0.1 or less. Sex, race, age, history of STD, and histories of alcohol, cocaine and methadone use were found to have p values of 0.1 or less and were therefore included in the analysis. Using SPSS, odds ratios and confidence intervals for each of these variables were obtained. A binary logistic regression was then performed, which generated adjusted odds ratios for each of these variables. Attributes of the anti-HCV positive and anti-HCV negative cohorts were compared, using a Chi squared test in SPSS for categorical variables, and an independent group t test for age. As a final step, a time-to-event plot was created, and the cumulative hazard function was calculated in SPSS, using death dates from the social security death index. This study was approved by the Johns Hopkins Institutional Review Board.

## Results

The baseline demographics for the 20-year study cohort are shown in Table 1. There were 288 men and 156 women. The majority were African American (63 %). Of the 444 patients, 284 (63 %) were anti-HCV positive in 1991. A history of cocaine use and heroin use was correlated with anti-HCV positivity in 1991. These results are described in the initial study and the subgroups of the found-records are presented in Table 1 [1].

**Table 1:** Baseline demographics of cohort analyzed at 20 years

Number of patients	444	
Mean age in 1991	33.7	SD 7.86

	Number	%
Gender		
Male	288	65
Female	156	35
Race		
White	118	19
Black	325	63
Hispanic	1	0.2
History IDU	301	68
Drugs of abuse		
Alcohol	300	68
Cocaine	315	71
Heroin	284	64
Methadone	39	9
Benzodiazepines	80	18
Marijuana	110	25
History of STD	175	39
HCV positive	284	64
HepBSAg positive	14	3
HepBSAb positive	142	32
HIV status		
HIV positive	46	10
HIV negative	149	34
HIV not tested	249	56
RPR positive	29	7

The results of the query of the Social Security Death Index are presented in Table 2, which compares the attributes of 20-year survivors to non-survivors. After 20 years, 82 of the 444 study participants were found to be deceased (18 %). The remaining patients were presumed to be alive. The mean ages of 20-year survivors and non-survivors in 1991 were 32.8 and 37.9, respectively (p < 0.001). Male patients had higher mortality than female patients, (23 vs. 10 %, p = 0.001) and white patients in the cohort had greater mortality than black patients (30 vs. 14 %, p < 0.001). Alcohol use (21 vs. 13 %, p = 0.05) and illicit methadone use (31 vs. 17 %, p = 0.04) in 1991 were more weakly associated with death at 20 years. Surprisingly, a history of sexually transmitted disease was more common among survivors.

**Table 2:** Mortality at 20 years, by subgroup

Demographic	Alive	Deceased	% deceased, %	P
Totals	362	82	18	
Age mean, years	32.8	37.9		<0.0001
Gender				
M	222	66	23	
F	140	16	10	0.001
Race*				
White	83	35	30	
Black	278	47	14	0.001
Hx IDU				
Yes	244	57	19	
No	118	25	17	0.72
Drugs of abuse				
Alcohol				
Yes	237	63	21	
No	125	19	13	0.05
Cocaine				
Yes	263	52	17	
No	99	30	23	0.1
Heroin				
Yes	233	51	18	
No	129	31	19	0.71
Methadone				
Yes	27	12	31	
No	335	70	17	0.04
Benzodiazepines				
Yes	61	19	24	
No	301	63	17	0.18
Marijuana				
Yes	90	20	18	
No	272	62	19	0.93
Hx STD				
Yes	152	23	13	
No	210	59	22	0.02
Hep C Ab				
Positive	228	56	20	
Negative	134	26	16	0.37
Hep BSAg				
Positive	10	4	29	
Negative	352	78	18	0.32
HepBSab				
Positive	115	27	19	
Negative	247	55	18	0.84
HIV				
Positive	23	7	23	
Negative*	339	74	18	0.46
RPR				
Positive	23	29	56	
Negative	339	415	55	0.75
AST				
<31	210	39	16	
>31 and <60	86	23	21	0.21
>60	66	20	23	
ALT				
<31	193	39	17	
>31 and <60	91	23	20	0.64
>60	78	20	20	

**Fig. 1:**
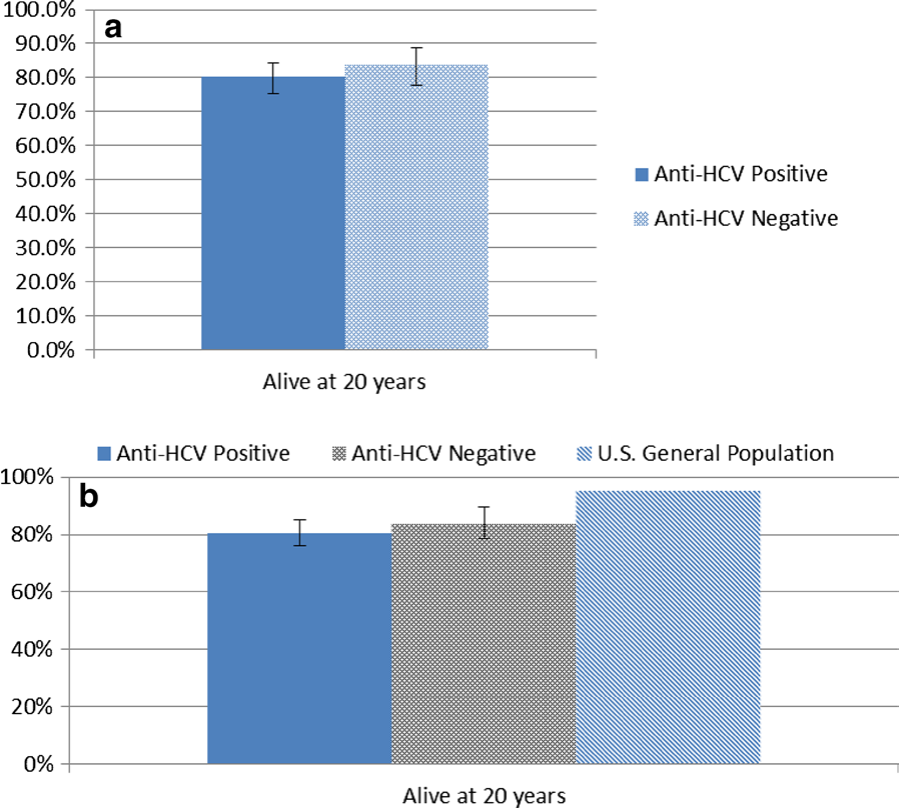
**a** Anti-HCV positivity did not confer a statistically significant 20-year mortality increase. **b** Comparison of deaths among IV drug users, compared to US General Population

Hepatitis C antibody status from 1991 was compared between 20-year survivors and non-survivors. Patients who were anti-HCV positive in 1991 had a 20 % mortality, while anti-HCV negative patients had a 16 % mortality. This difference was not statistically significant (p = 0.37), and is illustrated in Fig. 1a. These data are then shown in Fig. 1b in comparison with the expected survivorship of the US general population of the same age, using data from the 1993 CDC death table [26]. Hepatitis B surface antigen positivity and hepatitis B surface antibody positivity were also not associated with mortality differences, but sample sizes may have been too small in some cases to provide adequate power for the comparisons. Liver enzymes were recorded in the initial study in three ranges. Those with lower liver enzymes in 1991 had a slightly greater chance of survival, but this too did not reach statistical significance.

The HCV negative and HCV positive cohorts are compared in Table 3, to look for potential confounding variables. HCV positive patients were more likely to have been male, to have used cocaine and to have used illicit methadone. HCV positive patients were also older, on average, although HCV negative patients had a greater distribution of ages, including the youngest and oldest in the study.

**Table 3:** Attributes of hepatitis C negative and positive cohorts

	HCV negative (%)	HCV positive (%)	Total	*P* value*
Female	70 (0.45)	86 (0.55)	156	0.004
Male	90 (0.31)	198 (0.69)	288	
Nonwhite	123 (0.38)	203 (0.62)	326	0.217
White	37 (0.31)	81 (0.69)	118	
No alcohol	51 (0.35)	93 (0.65)	144	0.851
Alcohol	109 (0.36)	191 (0.64)	300	
No cocaine	66 (0.51)	63 (0.49)	129	<0.001
Cocaine	94 (0.3)	221 (0.7)	315	
No illicit methadone	152 (0.38)	253 (0.62)	405	0.034
Illicit methadone	8 (0.21)	31 (0.79)	39	
Did not report STD History	94 (0.35)	175 (0.65)	269	0.552
Reported STD history	66 (0.38)	109 (0.62)	175	
Mean age (years)	32.48	34.43	N/A	0.028**

* p value computed by Chi-square analysis

** p value computed by independent t test, equal variance not assumed

Factors associated with decreased 20-year survival are compared in Table 4, and their unadjusted odds ratios are shown. Results of a binary logistic regression are also included, and displayed as adjusted odds ratios. Male sex and a history of illicit methadone use remain statistically significant after regression analysis. Age also remains significant, with the odds of surviving decreasing slightly but significantly for each year of age of the individual at the start of the study. Again, hepatitis C positivity was not significantly associated with survival (OR 0.75, p = 0.29), and the non-significant association further diminished after binary logistic regression (AOR 0.87, p = 0.64).

**Table 4:** Regression analysis of hepatitis C viral status and factors associated with decreased 20-year survival

Factor	OR	CI (P value)	AOR	CI (P value)
Hepatitis C antibody (positive)	0.75	0.45–1.27 (0.29)	0.87	0.49–1.55 (0.64)
Sex (male)	0.39	0.22–0.71 (<0.01)	0.51	0.27–0.95 (0.03)
Age	0.93	0.31–0.96 (<0.01)	0.95	0.92–0.98 (<0.01)
Race (white)	0.39	0.24–0.64 (<0.01)	0.51	0.29–0.89 (0.02)
Alcohol use was present	0.58	0.33–1.02 (0.06)	0.76	0.41–1.4 (0.38)
Cocaine use was present	1.47	0.88–2.44 (0.14)	0.99	0.54–1.79 (0.96)
Illicit methadone use was present	0.46	0.22–0.96 (0.04)	0.46	0.21–0.99 (0.05)
History of sexually transmitted disease (true)	1.95	1.15–3.33 (0.01)	1.70	0.95–3.03 (0.07)

**Fig. 2:**
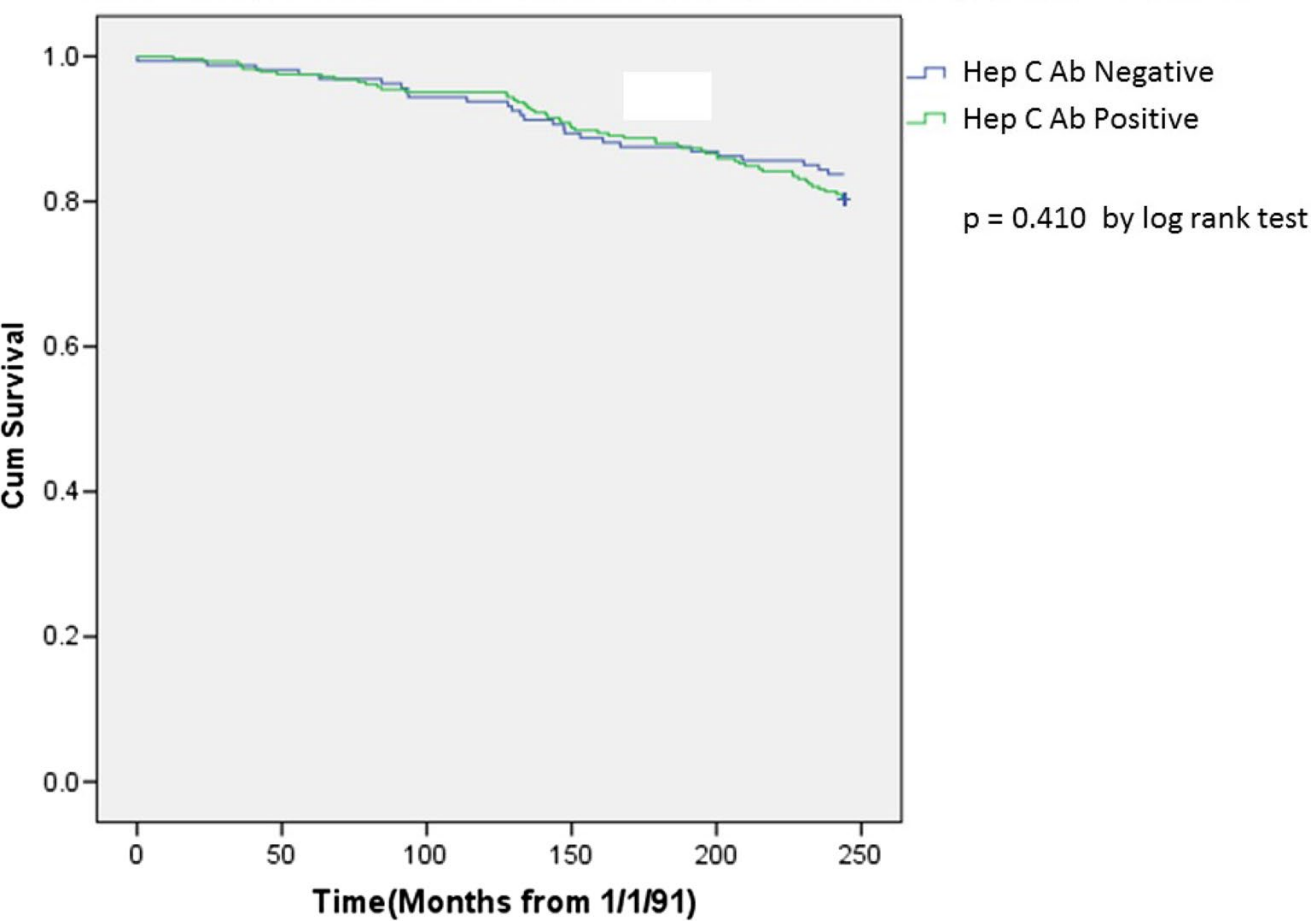
Time to event analysis of mortality for hepatitis C positive and negative patients

In a time to event analysis, the survival curves for HCV positive and HCV negative patients were essentially superimposable. The log rank test performed on the curve confirmed that the difference in the curves was not statistically significant (p = 0.410) (Fig. 2).

## Discussion

Our current study was remarkable for two findings. The first was the overall increased mortality rate of this cohort of patients with substance use disorders, as compared to that of the general population. The second finding was the lack of a statistical difference in mortality between those who were HCV positive in 1991, and those who were HCV negative at that time.

A query of the 1993 CDC mortality table shows that about 5000 out of 97,000 people aged 30–35 would have been expected to die in 20 years, a death rate of 5.1 % [26]. Our observed death rate in the study population was 18.4 %, indicating that the mortality rate of our cohort of patients with substance use disorders was substantially higher than that of the general US population. Our results are concordant with previous studies showing premature death rates among patients with injection drug use [25]. Of the drugs studied, only illicit methadone use appeared to decrease survival. Cocaine use made hepatitis C positivity more likely, but in concord with prior studies, showed no effect on survival [27].

The presence of the anti-HCV antibody in 1991 did not lead to a statistically significant increase in the 20-year mortality within this cohort. While our study did not have sufficient power to detect a small effect size, ample power was present for moderate or large effect sizes. It is therefore reasonable to conclude that the impact of hepatitis C infection on mortality, if present, was small. This study did not account for morbidity, so it is unknown whether cohort members were living with compensated or decompensated cirrhosis, or experienced other negative effects on their quality of life.

Our comparison of the attributes of the hepatitis C negative and hepatitis C positive cohorts shows that the hepatits C positive patients were more likely to have been male, to be older, and to have used illicit methadone, three factors which we would expect to make them more likely to die, given the data from this study. These attributes of the hepatitis C positive cohort are probably responsible for the diminished association of HCV positivity and mortality after the regression analysis. The fact that hepatitis C positive patients were more likely to have used cocaine likely made little difference in their survival. The fact that illicit use of methadone was associated with decreased survival may have been incidental but may also be related to the medication’s potential for respiratory depression and cardiac arrhythmias [28, 29]. Hazard from both of these factors may have been increased in patients using methadone outside of a supervised setting [30, 31].

Previous studies that have examined the mortality of patients with active hepatitis C virus tend to show little impact on mortality early in the disease course. Grady, et al. found 10-year survivals to be similar between seronegative and seropositive patients for the first 10 years, and suggested divergence around 20 years [32]. Gibson et al., similarly only showed increases in liver-related deaths past the 20 year mark, while Grebely et al. show the effect more pronounced only in patients over 50 years of age [33, 34]. Kieland et al. similarly show no change in mortality until three decades out from infection, in patients with an age greater than 50 [35]. Lee et al. documented a substantially greater hepatitis-C associated mortality, although their study did not focus specifically on patients with substance use disorders [36]. Our study serves to validate the emerging trend that hepatitis C positivity among patients with substance use disorders does not manifest a mortality difference for at least the first 20 years, and demonstrates this trend within an urban population in the United States.

The impact of hepatitis C virus specifically on patients with substance use disorders was studied by Evans et al. in San Francisco, CA, in a manner very similar to the one that we employed [37]. Similar to our results, they demonstrated that intravenous drug use itself was a risk factor for higher mortality; hepatitis C positivity only showed a non-significant trend towards higher mortality. Larney et al. studied hepatitis C related mortality among veterans with opioid use disorder and found no difference in overall mortality, although hepatitis C patients were more likely to a have a liver-related cause of death [38]. In an international study of three large national medical systems, Aspinal et al. document that both drug overdose and all-cause liver mortality were important contributors to mortality among patients with hepatitis C infection [39]. Hayashi et al. did a prospective study of patients with intravenous drug use in Vancouver, and did not find any increased liver mortality in the absence of HIV [40]. Several studies do indicate that HIV/HCV co-infection hastens the progression of liver disease [41–43].

Our study had several limitations. Cause of death was unavailable from the social security death index that we queried. As such, we could not make inferences about the frequency of liver-related mortality. At the time of the study in 1991, HCV RNA tests were not yet commonplace, and as such, there was no way to know which of the anti-HCV positive patients in the cohort had experienced spontaneous clearance. It is reasonable to assume that this would have constituted 10–15 % of the group. Another important limitation of this study is the fact that the HIV status of 56 % of the sample is unknown. Of those who were tested, 23 % were positive for HIV, making it a notable comorbidity within the cohort that could have accounted for some of its increased mortality rate as compared to the general population. Our study was also vulnerable to information bias, as it is possible that some of the cohort-members counted in the anti-HCV negative group may have contracted HCV over the 20 year period of the study. Conversely, some members of the anti-HCV positive group may have obtained HCV treatment. It is also possible that some of the patients were deceased but not reported to the Social Security administration, but this should not bias the results.

Our study has implications for health policy-related questions. Current medical practice has begun to shift attention toward stewardship and the allocation of medical resources, with campaigns such as *Choosing Wisely* gaining increased popularity [44]. Given a system with limited financial resources, it may be reasonable to question which therapies would provide the greatest health benefit to patients with substance use disorders. The average cost per treatment episode for outpatient substance use disorder is $2000–7000 [45]. The cost of new oral treatment for hepatitis C infection has been estimated at $84,000 per 12-week course of therapy [24]. Our study suggests that substance use disorders alone increase mortality, a finding that is consistent with prior research [46–48]. It does not, however, support the idea that hepatitis C virus was responsible for a significant fraction of that mortality. Although in a perfect system all patients would be treated for both, our study suggests that younger patients with substance use disorders may derive more mortality benefit from addiction treatment than they would derive from treatment of hepatitis C virus. This effect may shift over time as patients get older. Further research is necessary to establish which patients would be best served by treatment for one or both conditions.

## Conclusions

Hepatitis C antibody positivity was not associated with a statistically significant change in 20 years mortality among a cohort of patients with substance use disorders within an urban setting. The overall mortality of the cohort was higher than that of the general population in both hepatitis C positive and hepatitis C negative patients. The relative effect of hepatitis C status on mortality, if present, is likely quite small in comparison to the effect of substance use disorder within the population studied.
